# Supplementary material for: Spatial dysregulation of T follicular helper cells impairs vaccine responses in aging
Source: Nat Immunol. 2023 May 22;24(7):1124–37. doi: 10.1038/s41590-023-01519-9 (PMC10307630; doi:10.1038/s41590-023-01519-9)
Supplement: Supplementary file 1 — Supplementary Figs. 1–9 and Tables 1–5. [file 41590_2023_1519_MOESM1_ESM.pdf]

nature immunology

Article

https://doi.org/10.1038/s41590-023-01519-9

Spatial dysregulation of T follicular helper cells impairs vaccine responses in aging

In the format provided by the authors and unedited

Supplementary Information File

Supplementary Figure 1

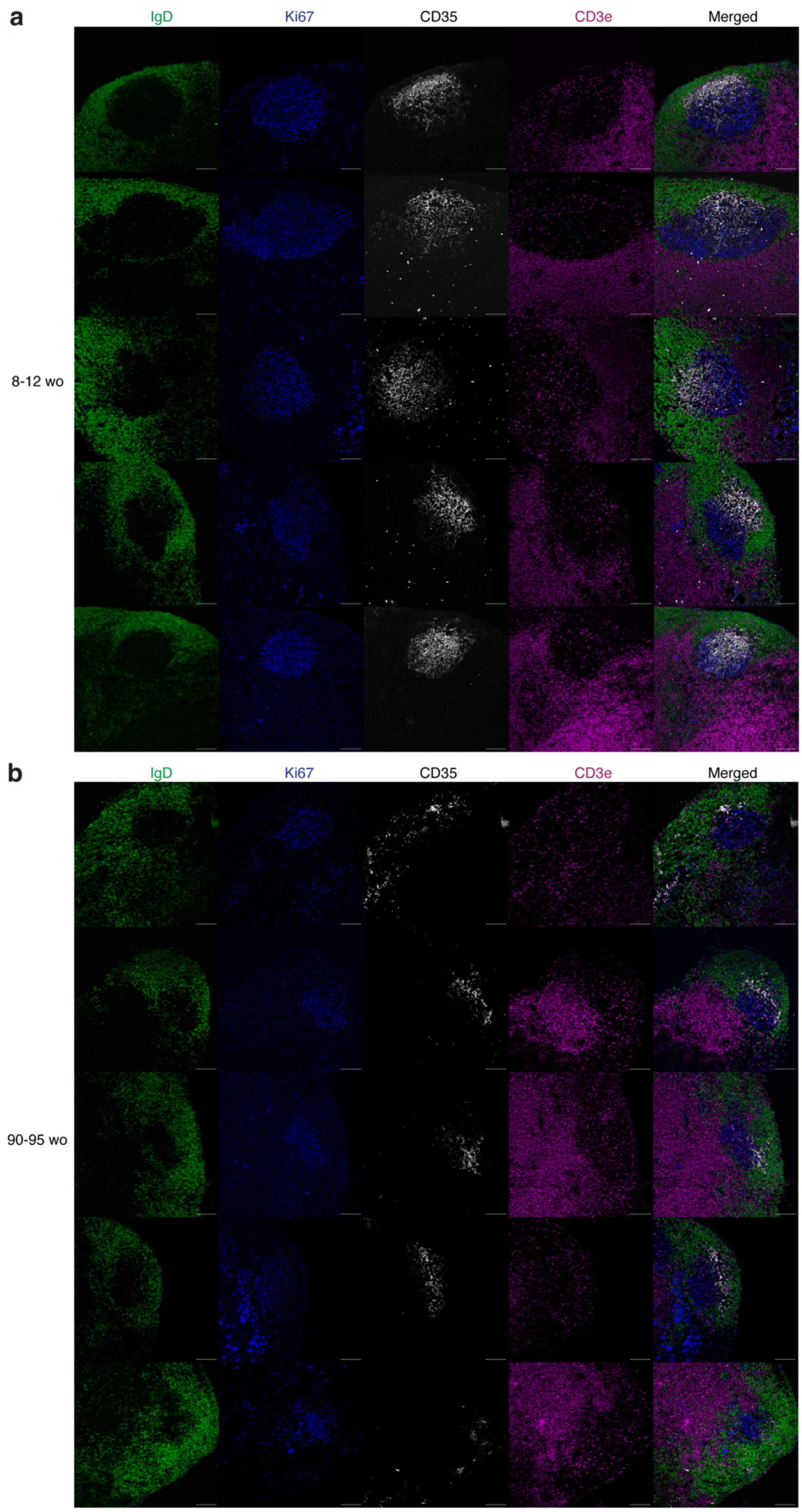

Supplementary Figure 1| Additional confocal images of GCs from iLNs of BALB/c
Additional representative confocal images of GCs from iLNs of different 8-12 week-old adult **(a)** and 90-95 week-old aged **(b)** BALB/c mice at 20x magnification where the scale bar is 100µm. Sections were stained for IgD (green), CD35 (white), Ki67 (blue) and CD3 (magenta). Data is representative of four independent experiments.

Supplementary Figure 2

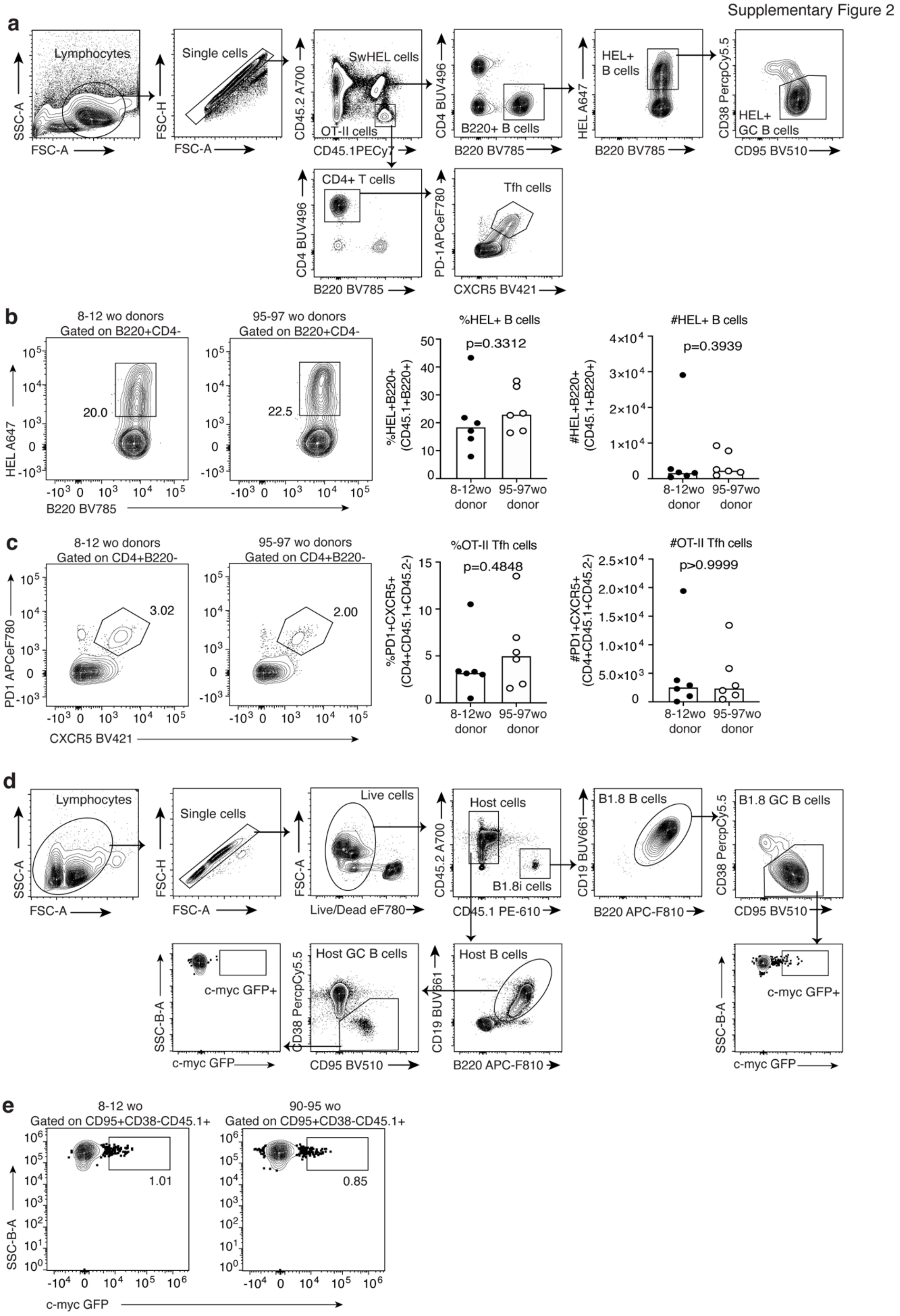

Supplementary Figure 2| Co-transfer of SW_{HEL} B cells and OT-II T cells into adult C57BL/6 mice and Adoptive transfer of B1-8i B cells into adult and aged C57BL/6 mice

(a) Gating strategy for the analysis of transferred HEL-binding SW_{HEL} B cells, SW_{HEL} derived GC B cells and OT-II derived Tfh cells in the iLNs of adult C57BL/6 recipient mice 10 days after immunisation with HEL-OVA in alum. **(b)** Representative flow cytometry plots and quantification of transferred HEL-binding B cells ($HEL^+B220^+CD45.1^+CD45.2^+$) in adult C57BL/6 recipients from adult and aged donors. **(c)** Representative flow cytometry plots and quantification of the OT-II derived Tfh cells ($PD1^+CXCR5^+CD4^+CD45.1^+CD45.2^-$) in adult C57BL/6 recipients from adult and aged donors. The values next to the gates on flow cytometry plots indicate the population percentage. The data is representative of three independent experiments (n=12) where each symbol represents a mouse, and the bar height represents the median. The p-values were obtained by performing an unpaired, two-tailed Mann-Whitney U test. **(d)** Gating strategy for identifying $c\text{-}Myc^+CD95^+CD38^-B220^+CD19^+CD45.1^+$ $B1\text{-}8^+$ germinal centre B cells after adoptive transfer into adult and aged mice and immunisation with NP-OVA in alum. **(e)** Representative flow cytometric contour plots of c-myc GFP expression in B1-8i derived GC B cells in young or aged mice. The values next to the gates on flow cytometry plots indicate the population percentage.

Supplementary Figure 3

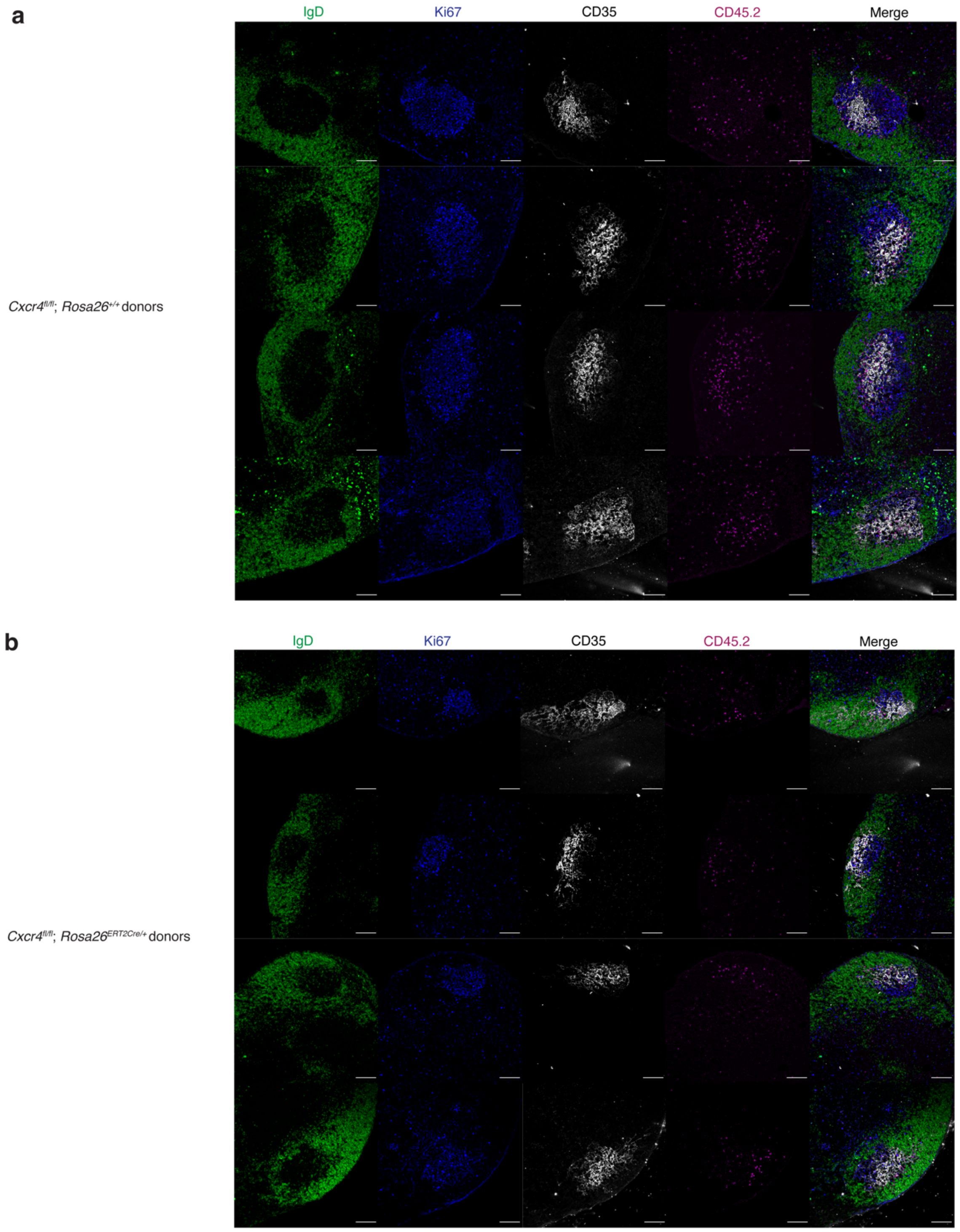

Supplementary Figure 3| Deletion of CXCR4 on T cells determines their location within the GC. Additional representative confocal images of the GCs from the iLNs of B6SJL mice which received tamoxifen-treated OT-II cells from either *Cxcr4*$^{fl/fl}$*; Rosa26*$^{+/+}$ **(a)** or *Cxcr4*$^{fl/fl}$*; Rosa26*$^{ERT2Cre/+}$ **(b)** mice; scale bars are 100μm. LN sections were stained for IgD (green), CD35 (white), Ki67 (blue) and CD45.2 (magenta). The data are representative of two independent experiments.

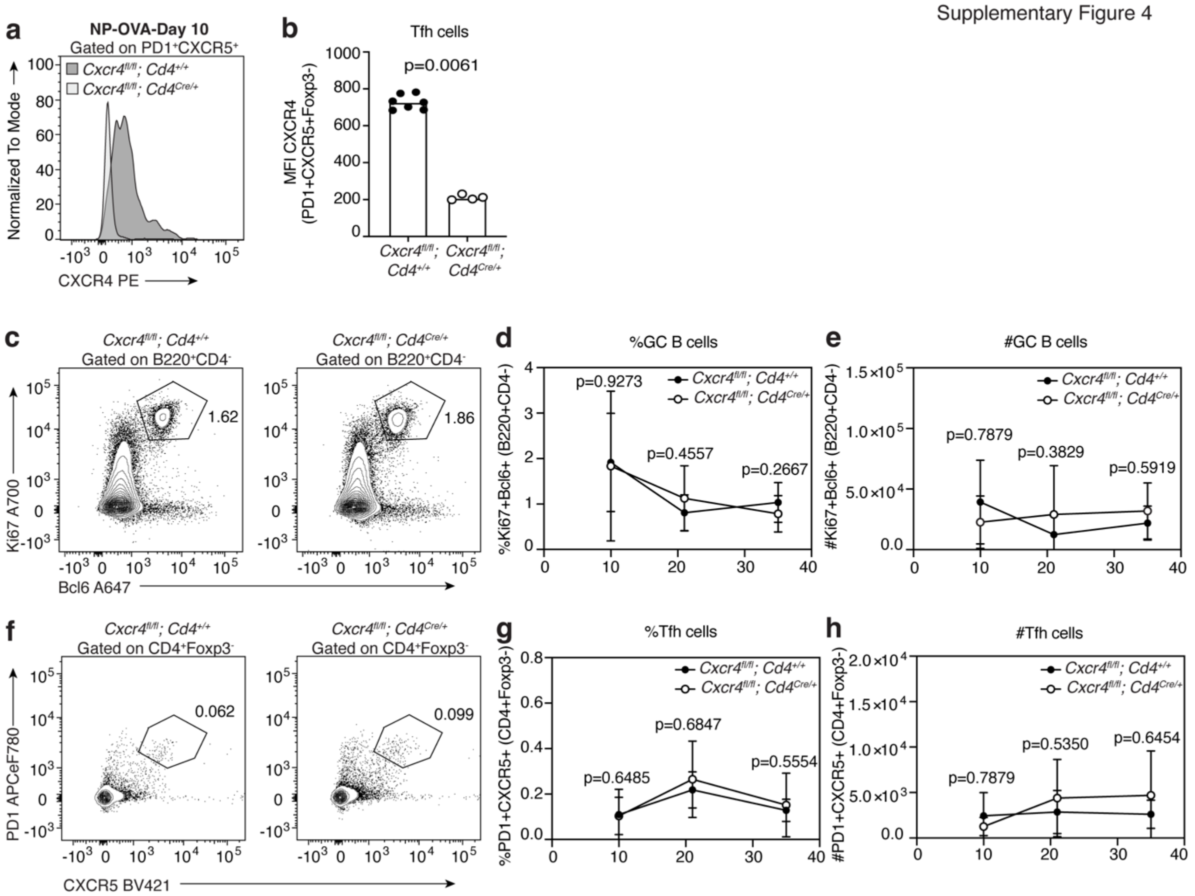

Supplementary Figure 4| CD4^{+} T cell-specific deletion of CXCR4, flow cytometric analysis of immunised *Cxcr4*$^{fl/fl}$; *Cd4*$^{Cre/+}$ mice

(a) Representative flow cytometry plot indicating the expression of CXCR4 by PD1^{+}CXCR5^{+}Foxp3^{-} Tfh cells in iLNs of *Cxcr4$^{fl/fl}$; Cd4$^{+/+}$* or *Cxcr4$^{fl/fl}$; Cd4$^{Cre/+}$* mice 10 days after NP-OVA immunisation. **(b)** Median fluorescence intensity (MFI) of CXCR4 by PD1^{+}CXCR5^{+}Foxp3^{-} Tfh cells of *Cxcr4$^{fl/fl}$; Cd4$^{+/+}$* or *Cxcr4$^{fl/fl}$; Cd4$^{Cre/+}$* mice. Bar height on graphs is indicative of the median, each symbol represents a mouse and p-values were obtained by performing an unpaired, two-tailed Mann-Whitney U test. The data are representative of two independent experiments (n=11). **(c)** Representative flow cytometry plots identifying Ki67^{+}Bcl6^{+} GC B cells 10 days after NP-OVA immunisation of *Cxcr4$^{fl/fl}$; Cd4$^{+/+}$* (left) and *Cxcr4$^{fl/fl}$; Cd4$^{Cre/+}$* (right) mice; values adjacent to gates indicate percentages. Quantification of the percentage **(d)** and total number **(e)** of Ki67^{+}Bcl6^{+} GC B cells at days 10, 21 and 35 after NP-OVA immunisation of *Cxcr4$^{fl/fl}$; Cd4$^{+/+}$* and *Cxcr4$^{fl/fl}$; Cd4$^{Cre/+}$* mice. **(f)** Representative flow cytometry plots identifying PD1^{+}CXCR5^{+} Tfh cells 10 days after NP-OVA immunisation of *Cxcr4$^{fl/fl}$; Cd4$^{+/+}$* (left) and *Cxcr4$^{fl/fl}$; Cd4$^{Cre/+}$* (right) mice; values adjacent to gates indicate percentages. Quantification of the percentage **(g)** and total number **(h)** of PD1^{+}CXCR5^{+} Tfh cells at days 10, 21 and 35 after NP-OVA immunisation of *Cxcr4$^{fl/fl}$; Cd4$^{+/+}$* and *Cxcr4$^{fl/fl}$; Cd4$^{Cre/+}$* mice. The data is representative of two independent experiments (n=14) where each symbol represents the mean ± SD and p-values were generated by performing a two-way ANOVA with Sidak's multiple comparisons test.

Supplementary Figure 5

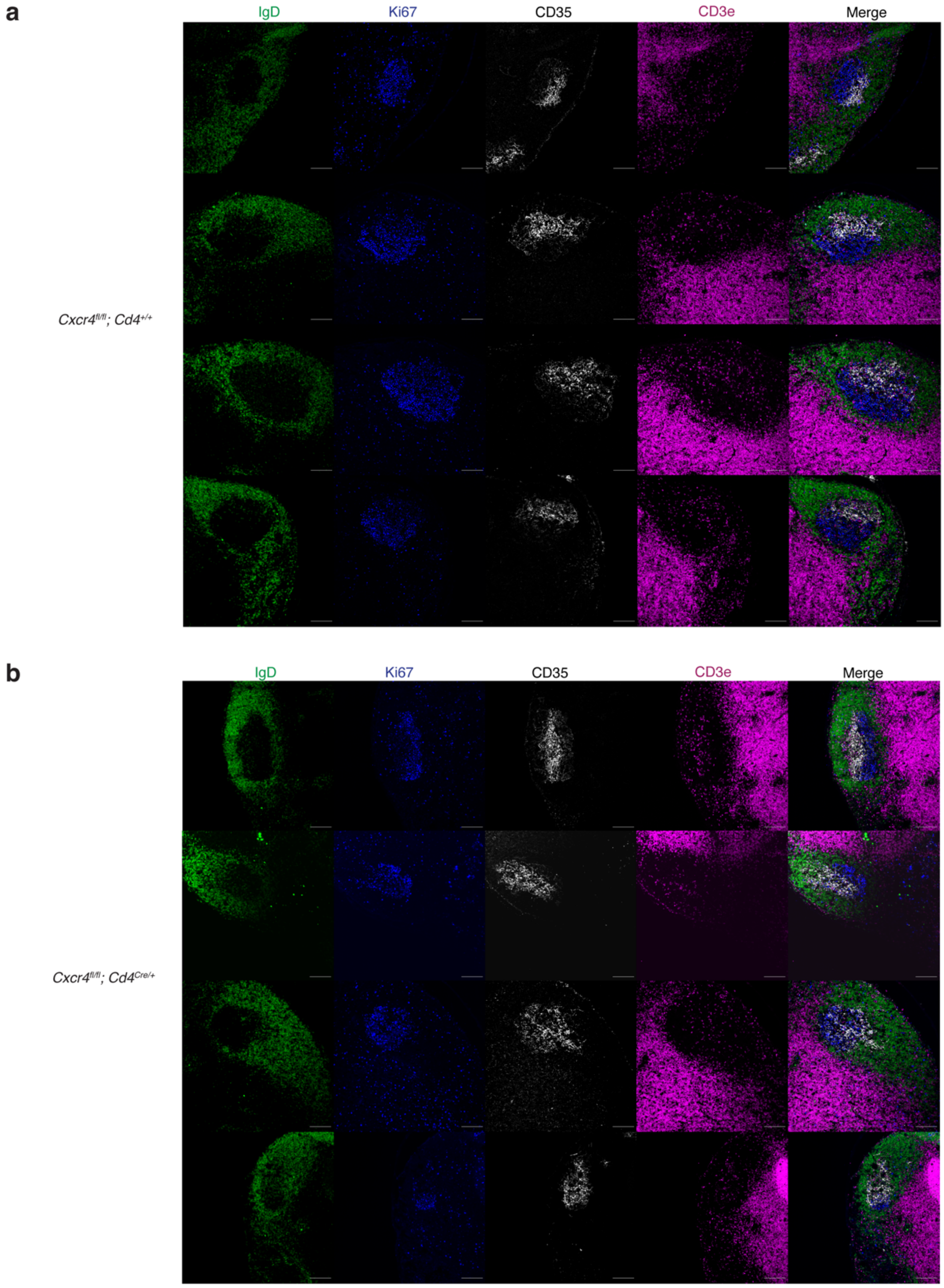

Supplementary Figure 5| Confocal imaging of immunised $\textit{Cxcr4}^{fl/fl}$**;** $\textit{Cd4}^{Cre/+}$ **mice**
Additional representative confocal images of GCs at day 10 post-immunisation with NP-OVA from iLNs of different control $\textit{Cxcr4}^{fl/fl}$; $\textit{Cd4}^{+/+}$ **(a)** and $\textit{Cxcr4}^{fl/fl}$; $\textit{Cd4}^{Cre/+}$ **(b)** mice. Images were taken at 20x magnification and the scale bars are 100µm. Sections were stained for IgD (green), CD35 (white), Ki67 (blue) and CD3e (magenta). Data is representative of two independent experiments.

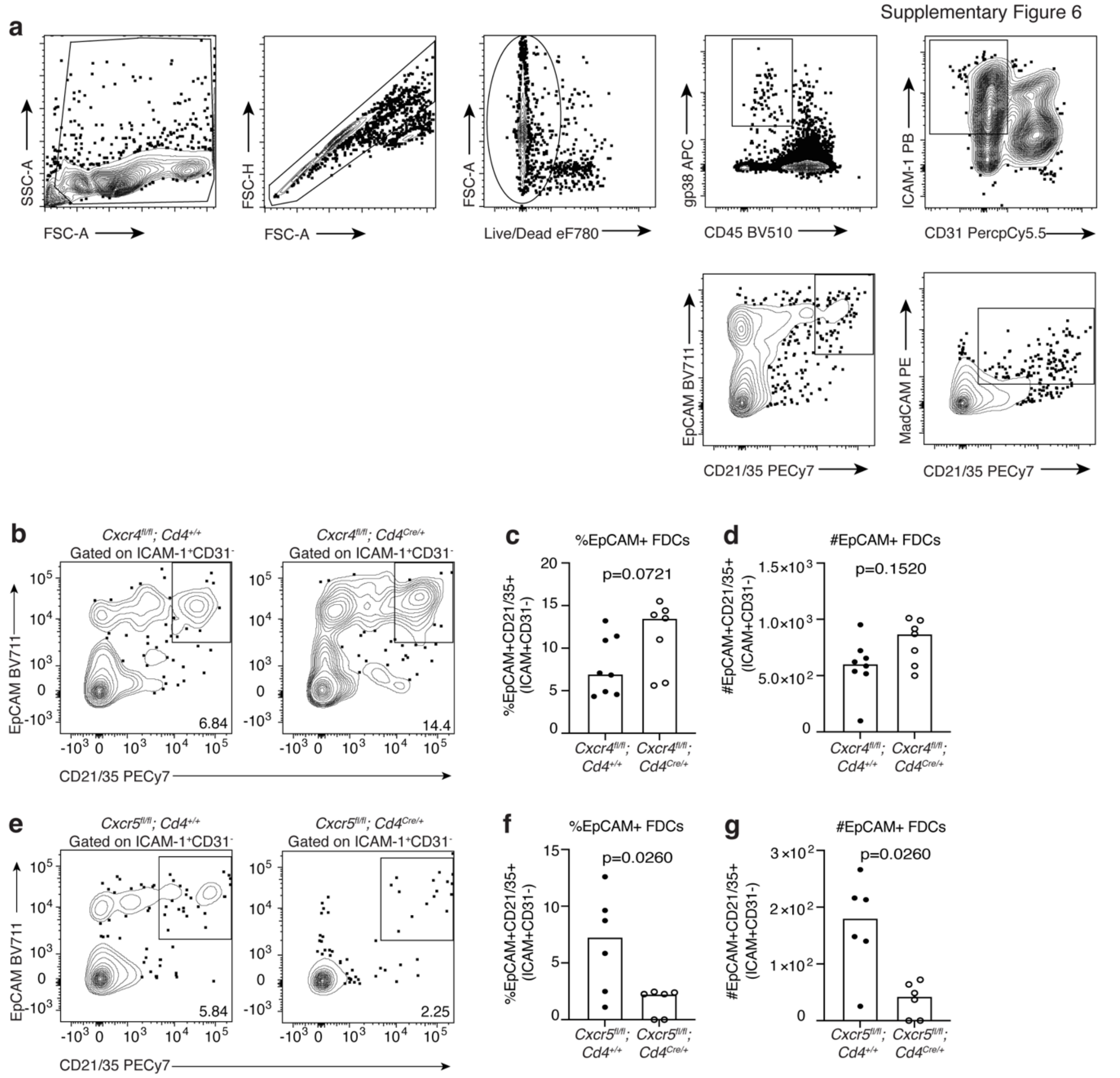

Supplementary Figure 6| Stromal cell staining in *Cxcr4*$^{fl/fl}$; *Cd4*$^{Cre/+}$ and *Cxcr5*$^{fl/fl}$; *Cd4*$^{Cre/+}$ mice

(a) Gating strategy for the analysis of MadCAM^{+}CD21/35^{+} and EpCAM^{+}CD21/35^{+} FDCs cells in *Cxcr4*$^{fl/fl}$; *Cd4*$^{Cre/+}$ and *Cxcr5*$^{fl/fl}$; *Cd4*$^{Cre/+}$mice 10 days after NP-OVA immunisation. **(b)** Representative flow cytometry plots indicating EpCAM+CD21/35+ FDCs in *Cxcr4*$^{fl/fl}$; *Cd4*$^{+/+}$ (left) and *Cxcr4*$^{fl/fl}$; *Cd4*$^{Cre/+}$ (right) mice. Quantification of the percentage **(c)** and total number **(d)** of EpCAM+CD21/35+ FDCs in *Cxcr4*$^{fl/fl}$; *Cd4*$^{+/+}$ and *Cxcr4*$^{fl/fl}$; *Cd4*$^{Cre/+}$ mice (n=15). **(e)** Representative flow cytometry plots indicating EpCAM+CD21/35+ FDCs in *Cxcr5*$^{fl/fl}$; *Cd4*$^{+/+}$ (left) and *Cxcr5*$^{fl/fl}$; *Cd4*$^{Cre/+}$ (right) mice. Quantification of the percentage **(f)** and total number **(g)** of EpCAM+CD21/35+ FDCs in *Cxcr5*$^{fl/fl}$; *Cd4*$^{+/+}$ and *Cxcr5*$^{fl/fl}$; *Cd4*$^{Cre/+}$ mice (n=12). Bar height on graphs is indicative of the median, each symbol represents a mouse and p-values were obtained by performing an unpaired, two-tailed Mann-Whitney U test. The data are representative of two independent experiments.

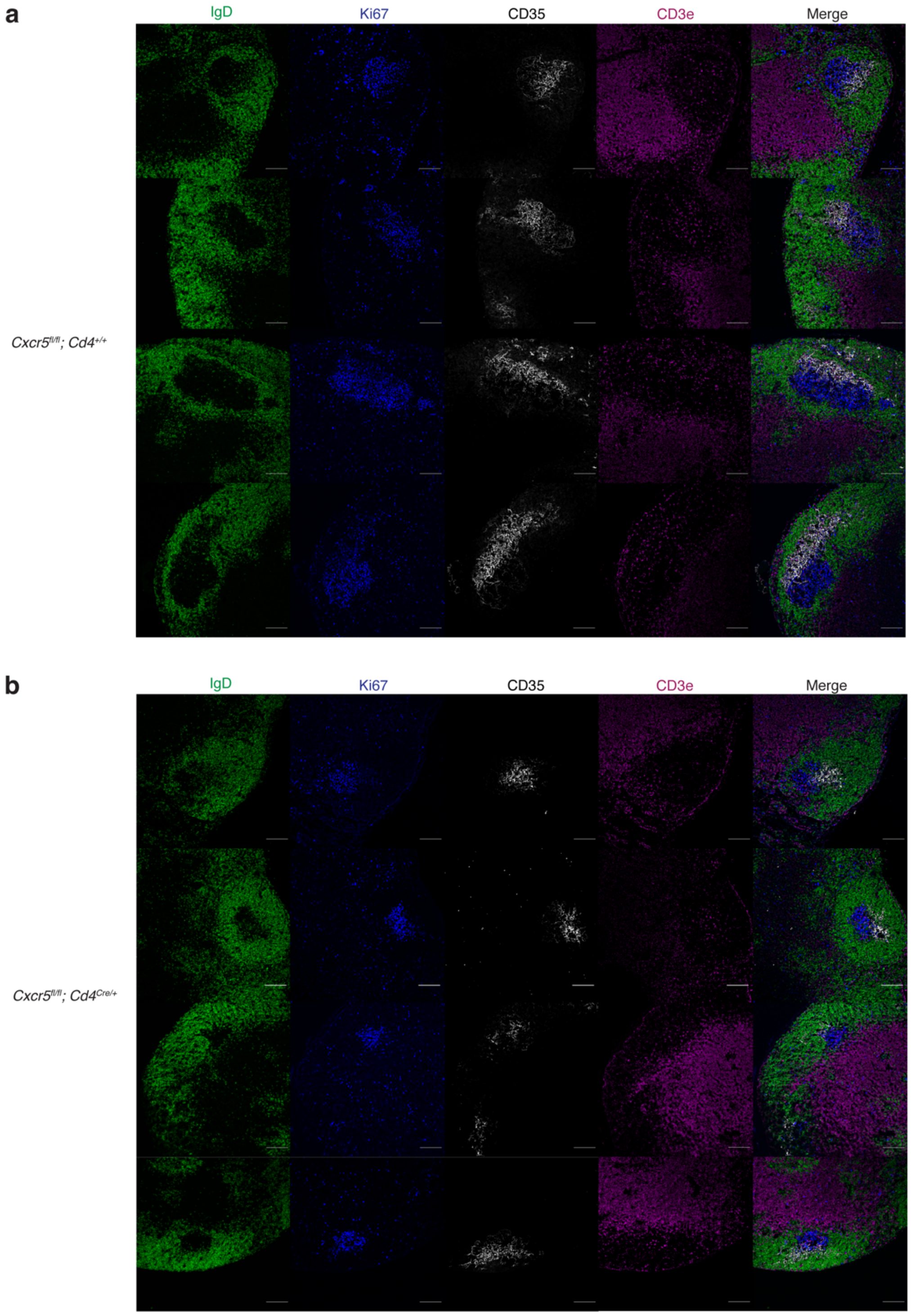

Supplementary Figure 7| Confocal images of immunised *Cxcr5*$^{fl/fl}$; *Cd4*$^{Cre/+}$ mice

Additional representative confocal images of GCs at day 10 post-immunisation with NP-OVA from iLNs of different control **(a)** *Cxcr5*$^{fl/fl}$; *Cd4*$^{+/+}$ or **(b)** *Cxcr5*$^{fl/fl}$; *Cd4*$^{Cre/+}$ mice. Images were taken at 20x magnification and the scale bars are 100µm. Sections were stained for IgD (green), CD35 (white), Ki67 (blue) and CD3e (magenta). Data is representative of two independent experiments.

Supplementary Figure 8

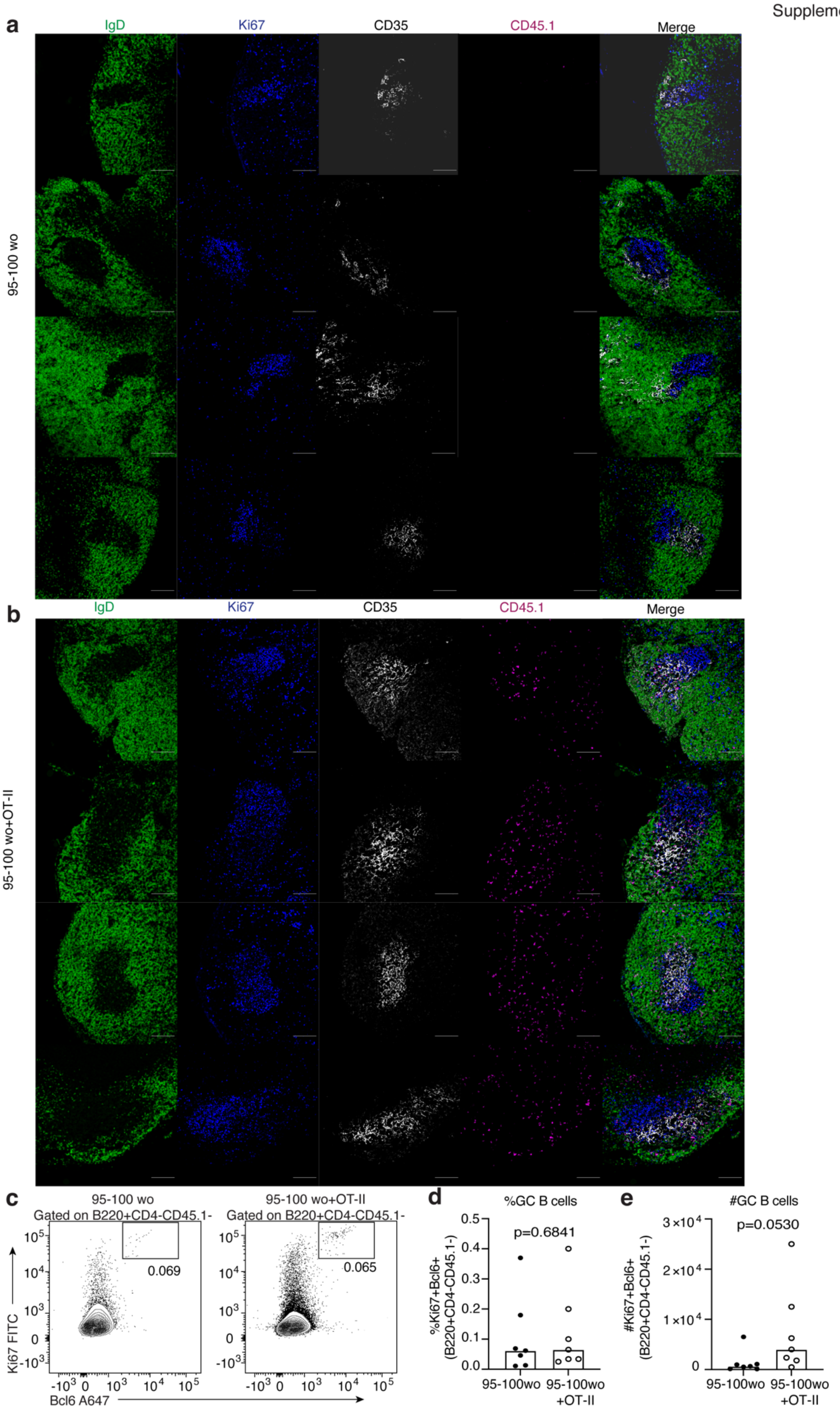

Supplementary Figure 8| Confocal images of iLNs from aged

Additional representative confocal images of GCs from iLNs of different 95-100 week-old aged C57BL/6 mice which received either **(a)** PBS or **(b)** purified OT-II cells from a younger adult OT-II mouse. Images were taken at 20x magnification where the scale bars are 100µm. Sections were stained for IgD (green), CD35 (white) and Ki67 (blue). **(c)** Representative flow cytometry plots and the percentage **(d)** and total number **(e)** of $Ki67^{+}Bcl6^{+}$ GC B cells in aged C57BL/6 mice, 7 days after NP-OVA immunisation, which received either an injection of PBS (left) or $CD4^{+}$ OT-II T cells (right); values adjacent to gates indicate percentage. Bar height on graphs indicates the median, each symbol represents a mouse (n=14), and p-values were obtained by performing an unpaired, two-tailed Mann-Whitney U test. Data are representative of two independent experiments.

a

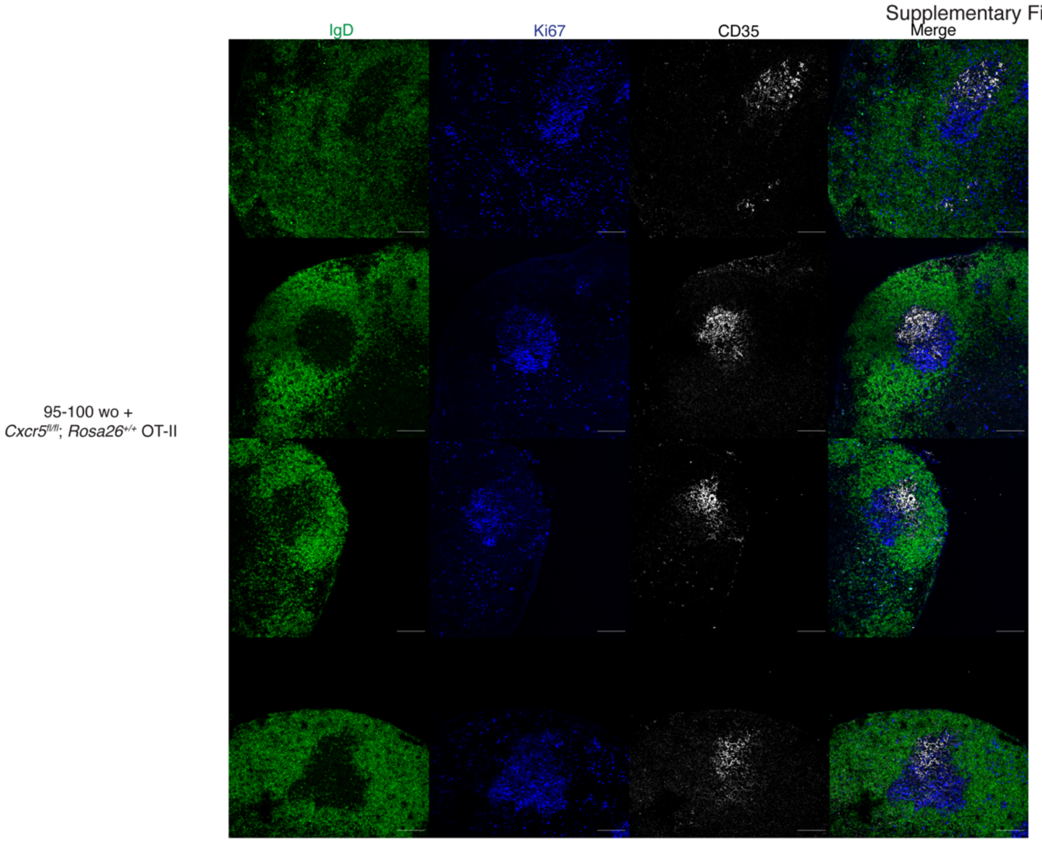
IgD
Ki67
CD35
Merge
95-100 wo +
Cxcr5fl/fl; Rosa26+/+ OT-II

b

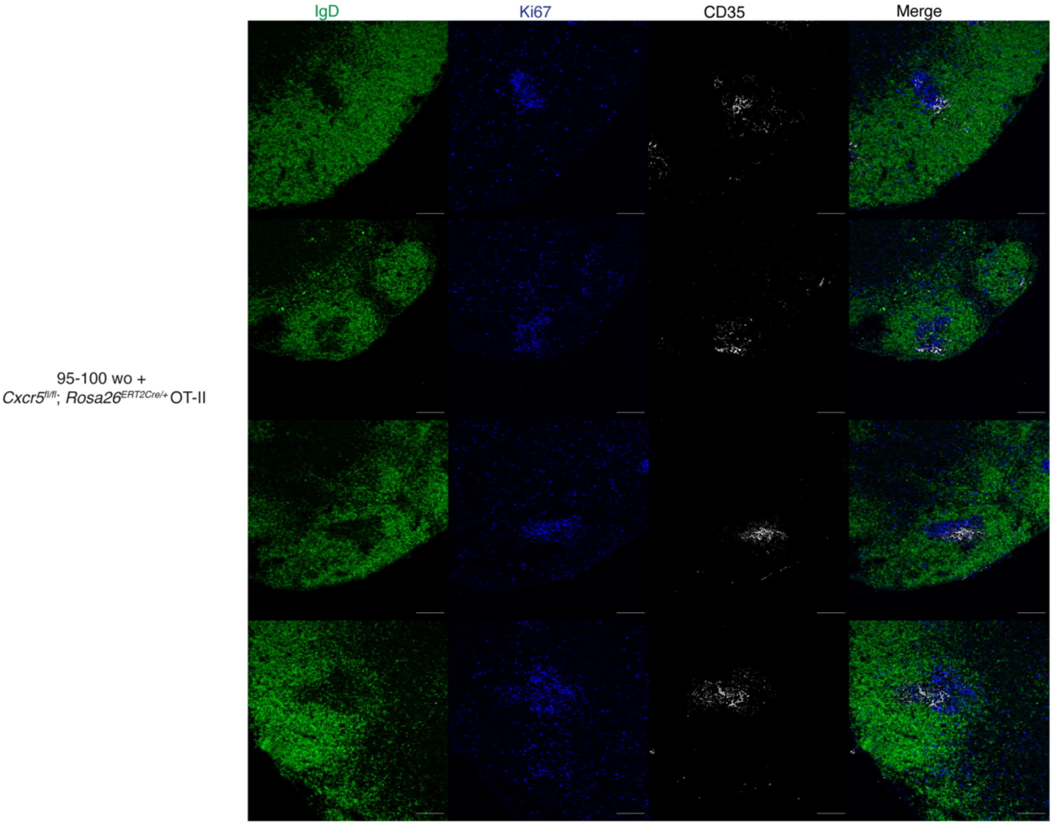
IgD
Ki67
CD35
Merge
95-100 wo +
Cxcr5fl/fl; Rosa26ERT2Cre/+ OT-II

Supplementary Figure 9| Additional confocal images of GCs from iLNs of aged C57BL/6 mice which received *Cxcr5*$^{fl/fl}$; *Rosa26*^{ERT2Cre} OT-II cells

Additional representative confocal images of GCs from iLNs of different 95-100 week-old aged C57BL/6 mice which received either **(a)** *Cxcr5*$^{fl/fl}$; *Rosa26*$^{+/+}$ OT-II cells or **(b)** *Cxcr5*$^{fl/fl}$; *Rosa26*$^{ERT2Cre/+}$ OT-II cells. Images were taken at 20x magnification where the scale bars are 100µm. Sections were stained for IgD (green), CD35 (white) and Ki67 (blue). Data is representative of two independent experiments.

Supplementary Table 1| Antibodies used for flow cytometric analysis of primary murine cells.

Antigen	Fluorochrome	Clone	Vendor
B220	APCFire810	RA3-6B2	BioLegend
B220	BV510	RA3-6B2	BioLegend
B220	BV785	RA3-6B2	BioLegend
Bcl-6	A647	K112-91	BD
Bcl-6	BV421	K112-91	BD
Bcl-6	PE-Cy7	K112-91	BD
CD4	APC	GK1.5	eBioscience
CD4	BUV395	GK1.5	BD
CD4	BUV496	GK1.5	BD
CD4	BV510	RM4-5	BioLegend
CD4	BV605	RM4-5	BioLegend
CD4	PE-Fire640	GK1.5	BioLegend
CD4	SV538	GK1.5	BioLegend
CD19	BUV661	1D3	BD
CD31	PerCPCy5.5	MEC13.3	BioLegend
CD38	eF450	90	eBioscience
CD38	PerCP-Cy5.5	90	eBioscience
CD44	BV510	IM7	BioLegend
CD44	PerCP-Cy5.5	IM7	BioLegend
CD45	BV510	30-F11	BioLegend
CD45.1	A700	A20	BioLegend
CD45.1	PE eFluor610	A20	eBioscience
CD45.1	PE-Cy7	A20	eBioscience
CD45.2	A700	104	eBioscience
CD45.2	PerCP-Cy5.5	104	eBioscience
CD54 (ICAM)	PacBlue	YN1/1.7.4	BioLegend
CD86	APC	GL-1	eBioscience
CD90.2	AF790	30-H12	BioLegend
CD95	BV510	Jo2	BD
CD95	PE-Cy7	Jo2	BD
CXCR4	APC	L276F12	BioLegend
CXCR4	BUV563	2B11	BD
CXCR4	PE	L276F12	BioLegend
CXCR4	PerCP-eF710	2B11	eBioscience
CXCR5	APC	L138D7	BioLegend
CXCR5	BV421	L138D7	BioLegend
CXCR5	BV785	L138D7	BioLegend
DEVED-FMK (Casp3)	FITC	-	eBioscience
EpCAM-1	BV711	G8.8	BioLegend
Foxp3	APC	FJK-16S	eBioscience
Foxp3	AF488	FJK-16s	eBioscience
Foxp3	PE-Cy5.5	FJK-16s	eBioscience
GL7	AF488	GL7	eBioscience
GR1	APC	RB6-8C5	eBioscience
Gp38	APC	8.1.1	BioLegend

HEL	A647	-	Conjugated in house
IgG1	BV605	A85-1	BD
IgM	APC	R6-60.2	BD
Ki67	AF700	SolA15	eBioscience
Ki67	APC eF780	SolA15	eBioscience
Ki67	FITC	SolA15	eBioscience
MadCAM-1	PE	MECA-367	BioLegend
NP	PE	-	Biosearch Technologies
CD21/35	PE-Cy7	eBio8D9	eBioscience
PD-1	FITC	RMP1-30	BioLegend
PD-1	APC eF780	J43	eBioscience
PD-1	BUV615	RMP1-30	BD
PD-1	PE-Cy7	RMP1-30	BioLegend
TCR Vα2	APC	B20.1	eBioscience
Cell viability dye	Aqua (525/50)	-	eBioscience
Cell Viability Dye	Blue (450/50)	-	eBioscience
Cell Trace Violet	eF450	-	eBioscience

Supplementary Table 2|Human antibodies and viability dye used for flow cytometric analysis

Antigen	Fluorochrome	Clone	Catalogue No.	Vendor
CXCR4	PE/Cy5	12GS	306507	BioLegend
CD45RA	SB570	F8-11-13	MCA88SBV570	BioRad
CD4	BUV496	M-T477	50175	BD Biosciences
CD3	SparkBlue550	SK7	344851	BioLegend
CD19	BUV615	HIB19	751273	BD Biosciences
Cell viability dye	ViaKrome808	-	C36628	Beckman Coulter

Supplementary Table 3| Primary antibodies used for immunofluorescence staining of lymph node sections

Species	Reactivity	Antigen	Conjugation	Clone	Vendor
Rat	Anti-mouse	IgD	FITC	11-26c.2a	BioLegend
Rat	Anti-mouse	IgD	AF647	11-26c.2a	BioLegend
Hamster	Anti-mouse	CD3e	APC	17A2	BioLegend
Hamster	Anti-mouse	CD3e	Purified	500A	Thermo Fisher
Rat	Anti-mouse	CD35	Biotin	8C12	BD
Rabbit	Anti-mouse	Ki67	Purified	Polyclonal	Abcam
Rat	Anti-mouse	Ki67	FITC	SolA15	Thermo Fisher
Rat	Anti-mouse	Ki67	eF450	SolA15	eBioscience
Rat	Anti-mouse	CD45.2	A700	104	eBioscience
Rat	Anti-mouse	CD45.1	BV605	A20	BioLegend
Mouse	-	IgG1 (isotype)	AF647	11711	R&D
Mouse	Anti-mouse/human	CXCL12/SDF-1	AF647	79018	R&D
Rat	Anti-mouse	CD21/35	Purified	7G6	BD
Rat	Anti-mouse	IgD	AF488	11-26	SouthernBiotech
Rat	Anti-mouse	IgD	AF488	11-26c.2a	Biolegend
Rat	Anti-mouse	CD16/32	BV421	190909	BD
Rat	Anti-mouse	CD4	Dylight550	GK1.4	BIOTREND
Rat	Anti-mouse/human	GL7	AF647	GL7	Biolegend

Supplementary Table 4| Secondary antibodies used for immunofluorescence staining of lymph node sections

Species	Reactivity	Antigen	Conjugation	Clone	Vendor
Goat	Anti-rabbit	IgG	AF568	Polyclonal	Thermo Fisher
Goat	Anti-rat	IgG	AF555	Polyclonal	Thermo Fisher
-	Biotin	-	Streptavidin AF568	-	Thermo Fisher
-	Biotin	-	Streptavidin BV421	-	BioLegend

Supplementary Table 5| List of parameters used for *in silico* modelling of the aged GC response

Parameter name	Value	Description
FDC number	200	Total number of FDCs
Percentage of FDC network	50	Percentage of the FDC network volume within the total GC volume
northweight	0.1	Tendency for Tc to stay in the light zone (polarity weight range from 0-1)
