# Supplementary material for: Spatial dysregulation of T follicular helper cells impairs vaccine responses in aging
Source: Nat Immunol. 2023 May 22;24(7):1124–37. doi: 10.1038/s41590-023-01519-9 (PMC10307630; doi:10.1038/s41590-023-01519-9)
Supplement: Supplementary file 2 — Reporting Summary [file 41590_2023_1519_MOESM2_ESM.pdf]

nature portfolio

Corresponding author(s): Michelle Linterman

Last updated by author(s): Apr 4, 2023

Reporting Summary

Nature Portfolio wishes to improve the reproducibility of the work that we publish. This form provides structure for consistency and transparency in reporting. For further information on Nature Portfolio policies, see our Editorial Policies and the Editorial Policy Checklist.

Statistics

For all statistical analyses, confirm that the following items are present in the figure legend, table legend, main text, or Methods section.

n/a	Confirmed	
☐	☒	The exact sample size (*n*) for each experimental group/condition, given as a discrete number and unit of measurement
☐	☒	A statement on whether measurements were taken from distinct samples or whether the same sample was measured repeatedly
☐	☒	The statistical test(s) used AND whether they are one- or two-sided<br>*Only common tests should be described solely by name; describe more complex techniques in the Methods section.*
☒	☐	A description of all covariates tested
☐	☒	A description of any assumptions or corrections, such as tests of normality and adjustment for multiple comparisons
☐	☒	A full description of the statistical parameters including central tendency (e.g. means) or other basic estimates (e.g. regression coefficient) AND variation (e.g. standard deviation) or associated estimates of uncertainty (e.g. confidence intervals)
☐	☒	For null hypothesis testing, the test statistic (e.g. *F*, *t*, *r*) with confidence intervals, effect sizes, degrees of freedom and *P* value noted<br>*Give P values as exact values whenever suitable.*
☒	☐	For Bayesian analysis, information on the choice of priors and Markov chain Monte Carlo settings
☒	☐	For hierarchical and complex designs, identification of the appropriate level for tests and full reporting of outcomes
☒	☐	Estimates of effect sizes (e.g. Cohen's *d*, Pearson's *r*), indicating how they were calculated

Our web collection on statistics for biologists contains articles on many of the points above.

Software and code

Policy information about availability of computer code

Data collection	For collection of flow cytometry data the following software was used: BD FACSDiva Software v9.0 and SpectroFlo Software v3.0<br>For acquisition of confocal microscopy images Zen Microscopy Software v3.2-3.5 was used.<br>For acquisition of ELISPOT images the ImmunoSpot version 5.0 CTL Cellular Technology Ltd Software was used.<br>For acquisition of raw ELISA absorbance values the PHERAstar FSX software v5.7 was used.
Data analysis	For analysis of flow cytometry data FlowJo v10.0 software was used.<br>For analysis and quantification of confocal images ImageJ (Fiji) v2.0.0-rc-69/1.52p and Cell Profiler v3.19 were used.<br>Graphs and statistics were generated using Graphpad Prism v6-9<br>Graphs and statistics for in silico modeling were generated using GLE v4.2<br>The code used for in silico modeling is available at: https://gitlab.com/simm/gc/hyphasma/-/releases/Denton2020<br>Analysis of W33L VH186.2 sequencing analysis was done using a custom pipeline on R v4.0.4<br>The code used for the W33L VH186.2 sequencing analysis is available at: https://github.com/lintermanlab/W33L_caller

For manuscripts utilizing custom algorithms or software that are central to the research but not yet described in published literature, software must be made available to editors and reviewers. We strongly encourage code deposition in a community repository (e.g. GitHub). See the Nature Portfolio guidelines for submitting code & software for further information.

Data

Policy information about availability of data

All manuscripts must include a data availability statement. This statement should provide the following information, where applicable:

- Accession codes, unique identifiers, or web links for publicly available datasets
- A description of any restrictions on data availability
- For clinical datasets or third party data, please ensure that the statement adheres to our policy

Data availability
Source data are provided with this paper.

Code availability
The code used for in silico modelling is available at: https://gitlab.com/simm/gc/hyphasma/-/releases/Denton2020. The code used for the W33L VH186.2 sequencing analysis is available at: https://github.com/lintermanlab/W33L_caller.

Human research participants

Policy information about studies involving human research participants and Sex and Gender in Research.

Reporting on sex and gender	Researchers were blinded to the sex or gender of research participants; the effect of sex or gender has not been investigated in this study.
Population characteristics	The study population is comprised of two comparison groups: 39 "aged" participants (between 66-98 years of age; mean age = 73.4) and 37 "young" samples (18 - 36 years of age; mean age = 26.5). 58% of participants self-identify as female. All participants were healthy, disease-naive adults.
Recruitment	Adult volunteers were recruited to this study on the basis of age. Peripheral blood was collected from healthy UK adults recruited through the NIHR Bioresource prior to and seven days after the seasonal influenza vaccine26,77. Samples were collected between October-February 2014-15 and October-December 2016, n = 37 participants 18–36 years old, n = 39 participants 66–98 years old. Volunteers were recruited through the NIHR BioResource Cambridge, which provided the infrastructure for the screening, recruitment and collection of these samples. Participants identified by NIHR BioResource Cambridge were sent an information sheet and asked to indicate their willingness to participate, as per their usual procedure. Written and informed consent was collected and retained by NIHR BioResource Cambridge. We are not aware of any biases, self-selected or otherwise, that may impact the results presented here.
Ethics oversight	Human samples were collected in accordance with the latest revision of the Declaration of Helsinki and the Guidelines for Good Clinical Practice (ICH-GCP). Informed consent was obtained from all participants. The samples collected with UK local research ethics committee approval (NRES Committee South Central - Hampshire A, REC reference 14/SC/1077), using the facilities of the NIHR Cambridge Bioresource (REC reference 04/Q0108/44).

Note that full information on the approval of the study protocol must also be provided in the manuscript.

Field-specific reporting

Please select the one below that is the best fit for your research. If you are not sure, read the appropriate sections before making your selection.

☒ Life sciences ☐ Behavioural & social sciences ☐ Ecological, evolutionary & environmental sciences

For a reference copy of the document with all sections, see nature.com/documents/nr-reporting-summary-flat.pdf

Life sciences study design

All studies must disclose on these points even when the disclosure is negative.

Sample size	All mouse experiments were performed with 4-10 mice per group and for aging studies a minimum of 5 aged mice per group were used. Sample size was chosen based on our previous experience working with genetically modified and aged mice, as well as the availability of aged mice or mice of appropriate genotypes.
Data exclusions	All data points were analysed including outliers unless there were technical errors. Any aged mice with lymphoma and/or solid tumors were also excluded from analysis.
Replication	All experiments were successfully replicated 2-4 times, the data shown is representative of these replicates.
Randomization	Where possible, mice were randomly allocated into aged and sex-matched experimental groups by staff of the Babraham Institute Biological Services Unit. For human blood samples, participants were recruited to the study on the basis of age aiming to recruit an equal number of

	younger and older adults, and samples coded by the NIHR BioResource and analysed in the laboratory. All available samples were used for this study.
Blinding	For experiments with aged mice, blinding was not possible due to phenotypic differences between adult and aged mice. For experiments with human blood samples, samples were coded by the NIHR BioResource and run blinded. Unblinding was done after data analysis to enable statistical comparisons.

Reporting for specific materials, systems and methods

We require information from authors about some types of materials, experimental systems and methods used in many studies. Here, indicate whether each material, system or method listed is relevant to your study. If you are not sure if a list item applies to your research, read the appropriate section before selecting a response.

Materials & experimental systems

n/a	Involved in the study
☐	☒ Antibodies
☒	☐ Eukaryotic cell lines
☒	☐ Palaeontology and archaeology
☐	☒ Animals and other organisms
☒	☐ Clinical data
☒	☐ Dual use research of concern

Methods

n/a	Involved in the study
☒	☐ ChIP-seq
☐	☒ Flow cytometry
☒	☐ MRI-based neuroimaging

Antibodies

Antibodies used

B220 APCFire810 RA3-6B2 BioLegend Cat#103278 Lot#B323073 (1/400)
B220 BV510 RA3-6B2 BioLegend Cat#103247 Lot#B2603705 (1/400)
B220 BV785 RA3-6B2 BioLegend Cat# 103246 Lot#B338778 (1/400)
Bcl6 A647 K112-91 BD Cat# 561525 Lot#9277097 (1/100)
Bcl-6 BV421 K112-91 BD Cat#563363 (1/100)
Bcl-6 PE-Cy7 K112-91 BD Cat# 563582 Lot#9050793 (1/100)
CD4 APC GK1.5 eBioscience Cat#17-0041-83 Lot#E07038-1635 (1/100)
CD4 BUV395 GK1.5 BD Cat#563790 Lot#0066907 (1/1000)
CD4 BUV496 GK1.5 BD Cat#612952 Lot#0080998 (1/1000)
CD4 BV510 RM4-5 BioLegend Cat#100559 Lot#B252816 (1/200)
CD4 BV605 RM4-5 BioLegend Cat#100547 Lot#B2667915 (1/200)
CD4 PE-Fire640 GK1.5 BioLegend Cat#100482 Lot#B315086 (1/2000)
CD4 SV538 GK1.5 BioLegend Cat#100485 Lot#B329034 (1/2000)
CD19 BUV661 1D3 BD Cat#612971 Lot#1085567 (1/2000)
CD31 PercpCy5.5 MEC13.3 BioLegend Cat#102522 Lot#B284200 (1/100)
CD38 eF450 90 eBioscience Cat# 48-03-81-82Lot#1934068 (1/100)
CD38 PercP-Cy5.5 90 eBioscience Cat# 102722 Lot#B341634 (1/1000)
CD44 BV510 IM7 BioLegend Cat#103044 Lot#B303963 (1/1000)
CD44 Percp-Cy5.5 IM7 BioLegend Cat#103032 Lot#B277590 (1/1000)
CD45 BV510 30-F11 BioLegend Cat#103138 Lot#B333193 (1/100)
CD45.1 A700 A20 BioLegend Cat#110724 Lot#B254605 (1/100)
CD45.1 PE eFluor610 A20 eBioscience Cat#61-0453-82 Lot#2024809 (1/100)
CD45.1 PE-Cy7 A20 eBioscience Cat#25-0453-82 Lot#2055156 (1/100)
CD45.2 A700 104 eBioscience Cat#56-0454-82 Lot#2123867 (1/100)
CD45.2 Percp-Cy5.5 104 eBioscience Cat#45-0454-82 Lot#4336370 (1/100)
CD54 (ICAM) PacBlue YN1/1.7.4 BioLegend Cat#116116 Lot#B241838 (1/100)
CD86 APC GL-1 eBioscience Cat#17-0862-82 Lot#19954468 (1/100)
CD90.2 AF790 30-H12 BioLegend Conjugated in-house (1/1000)
CD95 BV510 Jo2 BD Cat#563646 Lot#9325255 (1/1000)
CD95 PE-Cy7 Jo2 BD Cat#557653 Lot#7174919 (1/1000)
CXCR4 APC L276F12 BioLegend Cat#146508 Lot#B278598 (1/200)
CXCR4 BUV563 2B11 BD Cat#741313 Lot#1302258 (1/200)
CXCR4 PE L276F12 BioLegend Cat#146506 Lot#B242855(1/200)
CXCR4 Percp-eF710 2B11 eBioscience Cat#46-9991-82 Lot#2139606 (1/200)
CXCR5 APC L138D7 BioLegend Cat#145506 Lot#B270112 (1/100)
CXCR5 BV421 L138D7 BioLegend Cat#145512 Lot#B281252 (1/100)
CXCR5 BV785 L138D7 BioLegend Cat#145523 Lot#B310538 (1/100)
EpCAM-1 BV711 G8.8 BioLegend Cat#118233 Lot#B306636 (1/100)
Foxp3 APC FJK-16S eBioscience Cat#17-5773-82 Lot#1984797 (1/400)
Foxp3 AF488 FJK-16s eBioscience Cat#53-5773-82 Lot#1931448 (1/400)
Foxp3 PE-Cy5.5 FJK-16s eBioscience Cat#35-5773-82 Lot#2248878 (1/400)
GL7 AF488 GL7 eBioscience Cat#53-5902-82 Lot#2312432 (1/1000)
GR1 APC RB6-8C5 eBioscience Cat#56-5931-80 Lot#1920394 (1/100)
Gp38 APC 8.1.1 BioLegend Cat#127410 Lot#B268805 (1/100)
IgG1 BV605 A85-1 BD Cat#563285 Lot#0325234 (1/400)

IgM APC R6-60.2 BD Cat#562032 (1/100)
Ki67 AF700 SolA15 eBioscience Cat#56-5698-82 Lot#2261476 (1/1000)
Ki67 APCeF780 SolA15 eBioscience Cat#47-5698-82 (1/1000)
Ki67 FITC SolA15 eBioscience Cat#11-5698-82 Lot#2191034 (1/1000)
MadCAM-1 PE MECA-367 BioLegend Cat#120709 Lot#B299430 (1/100)
CD21/35 PE-Cy7 eBio8D9 eBioscience Cat#25-0211-82 Lot#2284271 (1/1000)
PD-1 FITC RMP1-30 BioLegend Cat#11-9981-81 Lot#2009760 (1/1000)
PD-1 APC eF780 J43 eBioscience Cat#47-9985-82 Lot#2194336 (1/1000)
PD-1 BUV615 RMP1-30 BD Cat#752354 Lot#1130154 (1/1000)
PD-1 PE-Cy7 RMP1-30 BioLegend Cat#109110 Lot#B263885 (1/2000)
TCRVa2 APC B20.1 eBioscience Cat#17-5812-82 Lot# 2142951 (1/100)
Cell Trace Violet eF450 - eBioscience Cat#C34557 (1/1000)
NP PE - Biosearch Technologies Cat#N-5070-1 (1/100)
Cell viability dye Aqua (525/50) - Cat#L34957 eBioscience (1/1000)
Cell Viability Dye Blue (450/50) - Cat#L23105 eBioscience (1/1000)

IgD FITC 11-26c.2a BioLegend Cat#405704 Lot#B271924 (1/400)
IgD AF647 11-26c.2a BioLegend Cat#405708 Lot#B259275 (1/200)
CD3e APC 17A2 BioLegend Cat#100236 Lot#B321239 (1/200)
CD3e Purified 500A Thermo Fisher Cat#14-0033-85 Lot#E03452-1633 (1/200)
CD35 Biotin 8C12 BD Cat#553816 Lot#7200747 (1/400)
Ki67 Purified Polyclonal Abcam Cat#ab15580 (1/200)
Ki67 FITC SolA15 Thermo Fisher Cat#11-5698-82 Lot#2191034 (1/200)
Ki67 eF450 SolA15 eBioscience Cat#48-5698-83 Lot#1998365 (1/100)
CD45.2 A700 104 eBioscience Cat#56-0454-82 Lot#2123867 (1/200)
CD45.1 BV605 A20 BioLegend Cat#110738 Lot#B267743 (1/200)
IgG1 (isotype) AF647 11711 R&D Cat#IC002R Lot#AEIX0219031 (1/200)
CXCL12/SDF-1 AF647 79018 R&D Cat#FAB350R Lot#1561071 (1/200)
CD35 Purified 8C12 BD Cat#558768 Lot#7236858 (1/200)
IgD AF488 11-26 SouthernBiotech Cat#1120-30 Lot#G0815-M329B (1/200)
Goat anti-Rat IgG (H+L) Cross-Adsorbed Secondary Antibody, AF555 Polyclonal Thermo Cat# A-21434 Lot#2089884 (1/1000)
Goat anti-Rat IgG (H+L) Cross-Adsorbed Secondary Antibody, AF568 Polyclonal Thermo Fisher Cat#A11077 Lot#870966 (1/1000)
Streptavidin AF568 - Thermo Fisher Cat#S11226 Lot#2045314 (1/1000)
Streptavidin BV421 - BioLegend Cat#405225 Lot#B279623 (1/1000)
IgD AF488 11-26c.2a BioLegend Cat#405718 (1/200)
CD16/32 BV421 190909 BD Cat#562896 (1/200)
CD4 Dylight550 GK1.4 BIOTREND Cat#C1637-100 (1/200)
GL7 AF647 GL7 Biolegend Cat#144605 (1/200)

CXCR4 PE/Cy5 12G5 BioLegend Cat#306507 (1/200)
CD45RA SB570 F8-11-13 BioRad Cat#MCA88SBV570 (1/200)
CD4 BUV496 M-T477 BD Biosciences Cat#50175 (1/200)
CD3 SparkBlue550 SK7 BioLegend Cat#344851 (1/400)
CD19 BUV615 HIB19 BD Biosciences Cat#751273 (1/200)
Cell viability dye ViaKrome808 - Beckman Coulter Cat#C36628 (1/2000)

Validation

All antibodies were validated by the manufacturer, for the species and the specified application (flow cytometry or imaging) for which they were used in this study. Antibodies used for flow cytometry were titrated prior to use and fluorescence minus one (FMO) controls were included to determine true positive staining. Antibodies used for microscopy were also titrated for optimal staining and both FMO controls and isotype controls were included for validation.

Animals and other research organisms

Policy information about studies involving animals; ARRIVE guidelines recommended for reporting animal research, and Sex and Gender in Research

Laboratory animals

C57BL/6, BALB/c, OT-II TCR-Tg, B6.SJL, SwHEL BCR-Tg, B1.8i BCR-Tg, Cd4Cre/+, Rosa26ERT2Cre/+, Cxcr4flox/flox and Cxcr5flox/flox mice were bred and maintained at the Babraham Institute Biological Support Unit. Cxcr5flox/flox;Cd4ERT2Cre/+ mice were bred and maintained at the Core Facility Animal Models of the Biomedical Centre of LMU Munich. Mice were housed under pathogen-free conditions and were kept at an ambient temperature of ~19-21°C with 52% relative humidity. Once weaned, mice were kept in individually ventilated cages with 1-5 mice per cage and were fed CRM (P) VP diet (Special Diet Services) ad libitum.

Wild animals

The study did not involve wild animals

Reporting on sex

Experiments with aged C57BL/6 mice were conducted using males due to the limited availability of aged female mice. Experiments with aged BALB/c mice were conducted using females due to the limited availability of aged male mice. All other experiments using only adult mice were performed with both male and female mice.

Field-collected samples

The study did not involve samples collected from the field

Ethics oversight

All mouse experimentation was approved by the Babraham Institute Animal Welfare and Ethical Review Body. Animal husbandry and experimentation complied with European Union and United Kingdom Home Office legislation and local standards (PPL: P4D4AF812).

Experiments with Cxcr5flox/flox;Cd4ERT2Cre/+ animals were performed in accordance with European regulation and federal law of Germany, and approved by the Regierung von Oberbayern.

Note that full information on the approval of the study protocol must also be provided in the manuscript.

Flow Cytometry

Plots

Confirm that:

☒ The axis labels state the marker and fluorochrome used (e.g. CD4-FITC).

☒ The axis scales are clearly visible. Include numbers along axes only for bottom left plot of group (a 'group' is an analysis of identical markers).

☒ All plots are contour plots with outliers or pseudocolor plots.

☒ A numerical value for number of cells or percentage (with statistics) is provided.

Methodology

Sample preparation	For lymphocyte staining, single cell suspensions from inguinal LNs were prepared by mechanical disruption of the tissues through a 70µm mesh in 2% FBS in PBS. The cell number and viability of samples were acquired using a CASY TT Cell Counter (Roche). 5x106 cells were transferred and stained in 5ml FACS tubes or 96- well plates. Cells were stained with Live/Dead Fixable Blue Dead Cell Stain (#L23105 Invitrogen) diluted at 1:1000 in PBS and incubated for 10 min at 4°C. Surface antibody stains were performed for 30 min-2 hours at 4°C in Brilliant stain buffer (#563794 BD Biosciences) after which cells were washed with 2% FBS in PBS and fixed using the Foxp3/Transcription Factor Staining Buffer Set (#00-5323-00 eBioscience). The antibodies used for flow cytometry of primary mouse cells are listed in Table 1. For intracellular staining, cells were incubated for 1 hour at 4°C with anti-Foxp3, anti-Ki67 and anti-Bcl6 antibodies diluted in 1x Permeabilization buffer (#00-8333-56 eBioscience).<br>For stromal cell staining, single cell suspensions from inguinal LNs were prepared by enzymatic digestion with 0.2mg/ml Collagenase P (#11213865001 Sigma), 0.8mg/ml Dispase II (#4942078001 Sigma) and 0.1mg/ml DNase I (#10104159001 Sigma) in plain RPMI medium (#11875093 Gibco). The LN capsules were penetrated with fine needles and incubated in the digestion buffer at 37°C for 15min and tubes were inverted every 5min. LNs were then triturated with a 1ml pipetted tip and the supernatant containing the released cells was collected in ice-cold 2%FBS in PBS with 2mM EDTA. The remaining fragments were processed in two more rounds as described, triturating every 5min. The cell suspensions were then filtered through a 100µm mesh and stained with the appropriate antibody cocktail.
Instrument	Samples were acquired on either an LSR Fortessa (BD Biosciences) with stained UltraComp eBeads Compensation Beads (#01-2222-41 Invitrogen) as single colour compensation controls or on a Cytek Aurora Spectral Cytometer (Cytek) with stained cells as single colour compensation controls.
Software	Flow cytometry data was collected using the BD FACSDiva Software v9.0 for samples acquired on an LSR Fortessa (BD Biosciences). For samples acquired on a Cytek Aurora Spectral Cytometer (Cytek), data was collected using the SpectroFlo v3.0 Software. All flow cytometry data was analysed using FlowJo v10.0 software.
Cell population abundance	Lymphocytes that were quantified by flow cytometry from a single draining inguinal lymph node yielded between 3-6x10^3 Tfh cells, 0.5-2x10^3 CXCR4+ Tfh cells, 0.5-2x10^3 Tfr cells, 2-6x10^4 GC B cells in adult wild type mice at the peak of the germinal centre response, D10-14. For adoptive transfers of B1.8i cmyc-GFP cells into wild type mice, 0.5-1.5x10^3 c-myc+ GC B cells were recovered from two draining inguinal lymph nodes. For stromal cell stains, roughly 0.1-1.5x10^3 FDCs were identified from two draining inguinal lymph nodes.<br><br>For VH186.2 sequencing, 48 NP+IgG1+ GC B cells were single-sorted per mouse into 96 well plates. From a single lymph node a total of roughly 0.5-3x10^3 NP+IgG1+ GC B cells could be recovered. After nested PCR for VH186.2, DNA from the sorted cells was run on an agarose gel to assess purity. Roughly >80% of sorted samples resulted in a PCR product of the right size.
Gating strategy	Lymphocytes were gated based on their size by FSC-A/SSC-A after which Single Cells were gated based on either SSC-W/SSC-A or FSC-H/FSC-A. Live cells from experiments including live/dead stains were then gated based on being negative for the used live/dead stain against FSC-A. Lymphocytes were then separated into either CD4+B220- T cells or B220+CD4- B cells. CD4+B220- T cells were then further gated into either Foxp3+CD4+ T regulatory cells or Foxp3-CD4+ T conventional cells. Follicular T helper cells were gated as PD1+CXCR5+ cells from the Foxp3-CD4+ T conventional cells parent gate while Follicular T regulatory cells were gated as PD1+CXCR5+ cells from the Foxp3+CD4+ T regulatory cell parent gate. CXCR4+CD4+ Follicular helper T cells were gated using a Fluorescence Minus One (FMO) control. For experiments using mouse models of T cell-specific CXCR5 deletion, T follicular helper (Tfh) cells were also gated as PD1+Bcl6+ from a Foxp3-CD4+ T cell parent gate. To identify Germinal Centre (GC) B cells, either Ki67+Bcl6+, Bcl6+CD38-, CD38-GL7+ or CD38-CD95+ were gated from B220 +CD4- B cells. For adoptive transfer experiments, live cells were gated based on their expression of the congenic markers CD45.1 and CD45.2 to separate out transferred cells from host cells, after which GC B cells and Tfh cells were identified as described above. GC B cells were then further divided into either CXCR4hiCD86lo centroblasts, CD86hiCXCR4lo centrocytes and cmyc-GFP+ GC B cells.<br>For Stromal cells gating, cells were first gated based on their size by FSC-A/SSC-A after which Single Cells were gated based on FSC-H/FSC-A. Live cells were then gated based on being negative for the live/dead eF780 stain against FSC-A. From the live cells, mesenchyme stroma was then gated as gp38+CD45- to exclude lymphocytes. This population was then further gated into ICAM+CD31- after which Follicular Dendritic Cells were gated as either EpCAM+CD21/35+ or MadCAM+CD21/35+.

☒ Tick this box to confirm that a figure exemplifying the gating strategy is provided in the Supplementary Information.
